# Multiple myeloma cell-derived microvesicles are enriched in CD147 expression and enhance tumor cell proliferation

**DOI:** 10.18632/oncotarget.2159

**Published:** 2014-07-03

**Authors:** Bonnie K. Arendt, Denise K. Walters, Xiaosheng Wu, Renee C. Tschumper, Diane F. Jelinek

**Affiliations:** ^1^ Department of Immunology, Mayo Clinic, College of Medicine, Rochester, MN; ^2^ Division of Hematology, Department of Medicine, Mayo Clinic, College of Medicine, Rochester, MN

**Keywords:** MPT0G030, PKCδ, E-cadherin, HDAC, differentiation

## Abstract

Multiple myeloma (MM) is characterized by the clonal expansion of malignant plasma cells within the bone marrow. There is a growing literature that tumor cells release biologically active microvesicles (MVs) that modify both local and distant microenvironments. In this study, our goals were to determine if MM cells release MVs, and if so, begin to characterize their biologic activity. Herein we present clear evidence that not only do both patient MM cells and human MM cell lines (HMCLs) release MVs, but that these MVs stimulate MM cell growth. Of interest, MM-derived MVs were enriched with the biologically active form of CD147, a transmembrane molecule previously shown by us to be crucial for MM cell proliferation. Using MVs isolated from HMCLs stably transfected with a CD147-GFP fusion construct (CD147^GFP^), we observed binding and internalization of MV-derived CD147 with HMCLs. Cells with greater CD147^GFP^ internalization proliferated at a higher rate than did cells with less CD147^GFP^ association. Lastly, MVs obtained from CD147 downregulated HMCLs were attenuated in their ability to stimulate HMCL proliferation. In summary, this study demonstrates the significance of MV shedding and MV-mediated intercellular communication on malignant plasma cell proliferation, and identifies the role of MV-enriched CD147 in this process.

## INTRODUCTION

Multiple myeloma (MM) remains a largely incurable neoplasm defined by the presence of a clonal expansion of malignant plasma cells (PC) in the bone marrow (BM), ≥3 g/dL of monoclonal immunoglobulin (Ig; M-protein), and the presence of end organ damage which may include lytic bone lesions [1]. Monoclonal gammopathy of undetermined significance (MGUS) and smoldering (SMM) are the asymptomatic stages which precede MM, with MGUS being considered the premalignant stage [2]. Although the clonal PC populations in both asymptomatic conditions may remain remarkably stable for years, MGUS and SMM patients have a significant, life-long increased risk of progressing to overt MM at a rate of 1% and 10% per year, respectively [2, 3]. While there have been considerable advances in treatment of this disease, the median survival of MM patients still remains only 4-7 years [4].

Over the last decade, significant investigation in the MM field has demonstrated that the interaction of malignant PCs with BM stromal cells within the microenvironment is essential for survival of the PC clone [5, 6]. The observation of this intercellular communication and that MM cells modify the microenvironment is highly suggestive that one of the first biologically relevant changes necessary for progression of MGUS or SMM to MM is for a member(s) of the clone to evolve and acquire the molecular changes needed to reprogram its immediate microenvironment into a supportive, growth-promoting microenvironment. Microvesicles (MVs) are increasingly recognized as mediators of intercellular communication due to their capacity to merge with and transfer a collection of bioactive molecules to recipient cells [7]. MVs are cell-derived particles that are spontaneously shed by a variety of cell types under normal physiological conditions.[7] Notably, tumor cells have been shown to shed MVs in an increased, dysregulated [8, 9] fashion, resulting in the presence of circulating MVs in patients with various types of cancers [10, 11]. MVs range in size from 0.05−1.0 μm and formation occurs through the outward budding and fission of the plasma membrane which can be induced by multiple events including the activation of PKC and/or increased intracellular calcium (Ca^2+^) concentrations [12, 13]. Studies have shown that the molecular and genetic contents of MVs are very specific rather than a random sampling of all molecules expressed in the originating cell [14] and MV release is thought to be dynamic and dependent on both the cell type and activation status of the cell from which they are released [15]. Compelling evidence suggests a role for tumor-derived MV-mediated modulation of cells within the tumor microenvironment of various malignancies, including lung, breast, melanoma, and CLL [16-19]. Moreover, MV-modulation activity has been shown to correlate with tumor cell invasiveness resulting in angiogenesis and metastasis.

Despite the significant number of studies showing a role for TDMVs in various solid tumors, to date there has been very little study of TDMVs in MM. To our knowledge, there have been only three published studies of TDMVs and MM, and the scope in each case was extremely limited. One group studied the effect of serum deprivation on the size of MVs released by two HMCLs [20] and in a separate paper, examined the ability of MVs from one HMCL to stimulate endothelial cell proliferation [21]. The second group performed proteomic analysis of MVs released by two HMCLs and compared the profiles with intact cells [22]. Although these papers demonstrate MM cell release of MVs, current knowledge of the biological activity of MM cell derived MVs remains very limited.

Regarding a potential biological role for MM cell derived MVs, the CD147 molecule has been shown to be present on MVs isolated from lung and ovarian cancer cell lines [23, 24]. We were the first to show that CD147 is upregulated in MM cells and appears necessary for tumor cell growth. In addition, expression levels of CD147 increase during MM progression [25]. CD147, also known as extracellular matrix metalloproteinase (MMP) inducer (EMMPRIN), is a transmembrane glycoprotein ubiquitously expressed at a low level on the surface of many different cell types [26]. Increased CD147 expression is biologically relevant because of its ability to increase angiogenesis [27], tumor growth [28], and MMP production leading to extracellular matrix degradation [28, 29]. Based on these findings as well as the growing body of literature regarding the role of MVs in intercellular communication, the aim of the present study was to continue our research of CD147 by investigating its potential involvement in the disease as a constituent of MM cell released MVs. Our data clearly demonstrate that malignant PCs shed bioactive MVs, and that this bioactivity can partially be attributed to the presence of CD147 within these MVs.

## RESULTS

### Analysis of MM cell-derived MVs

**Figure 1:**
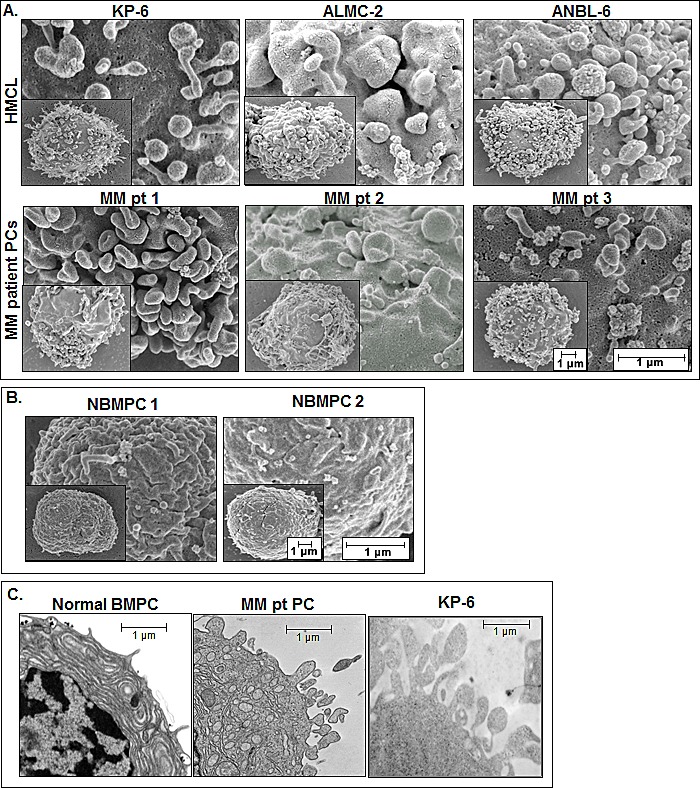
EM analysis of surface associated MVs A) MV production as revealed by SEM on 3 HMCLs and 3 primary patient CD138^+^ MM cell samples. Whole cell images are shown in the lower left of each panel and the scales for each image are shown in the bottom right panel (MM pt 3). B) SEM analysis of 2 normal bone marrow PC samples. C) Surface MV comparison by TEM of normal bone marrow PC (left), MM patient PC (middle), and the KP-6 HMCL (right).

Although cell-to-cell contact and subsequent secretion of soluble factors has long been known to facilitate communication within the tumor microenvironment, recent evidence suggests that these factors can also be released via membrane-bound specialized vesicles which contain cellular contents that aid intercellular communication. Although there is emerging evidence that HMCLs release MVs, it remains unknown if primary patient MM cells release MVs, and if so, does this differ from normal BM PCs. To address these questions, we first used SEM to analyze HMCLs, primary patient MM cells, and normal BM PCs for evidence of cell surface associated MVs. Figure 1A demonstrates that the surface of representative HMCLs was rich with MVs both spherical and villi-like in appearance and that primary patient MM PCs showed similar structures, effectively demonstrating MV projections around the cell membrane periphery. Moreover, the size of these surface structures ranged from ~0.05−1.0 μm in diameter in both HMCLs and patient MM cells and is therefore consistent with the published range of MVs [30-32]. In striking contrast, SEM analysis of normal BM PCs revealed a remarkably smooth cell surface (Figure 1B). We also used TEM and the results shown in Figure 1C support the SEM data, with normal BM PC cell surfaces exhibiting fewer MVs than MM PCs and the KP-6 HMCL.

**Figure 2:**
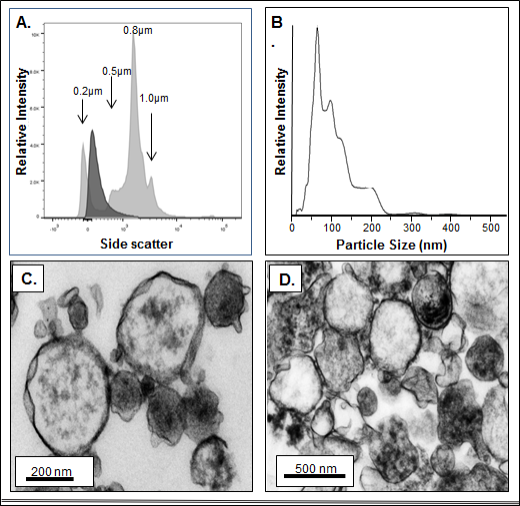
MM-derived MV dimensions A) Histogram showing by flow cytometry the size of ALMC-2 derived annexin-V+ MVs (dark gray histogram) in comparison with 0.2, 0.5, 0.8 and 1.0 μm calibration beads (light gray histograms). B) Nanoparticle tracking analysis of MVs isolated from purified MM patient plasma cells. C, D) TEM of RM43 and ALMC-2 HMCL MVs, respectively.

To verify that the apparent surface associated MVs were indeed being released we collected conditioned media from several HMCLs and processed it as described in the Materials and Methods. The size of the MVs was analyzed using several complementary methods. We first used flow cytometry and submicron sizing beads and the size of HMCL derived MVs was consistent with the reported size range of MVs (Figure 2A). Because it is known that some MVs are too small to be detected by flow cytometry [33], we also performed Nanoparticle Tracking Analysis to independently characterize the size of MM-derived MVs. Figure 2B shows a representative analysis of MV size using this technology and, as expected, this method permitted demonstration of a significant number of MVs too small to be detected by flow cytometry. Third, we also used TEM to assess size. Figures 2C and 2D demonstrate the spherical structure of MVs isolated from the HMCLs RM43 and ALMC-2, respectively. This analysis also demonstrates the range in size of MM cell derived MVs.

### MM-derived MVs are annexin-V+ and express full-length CD147

**Figure 3:**
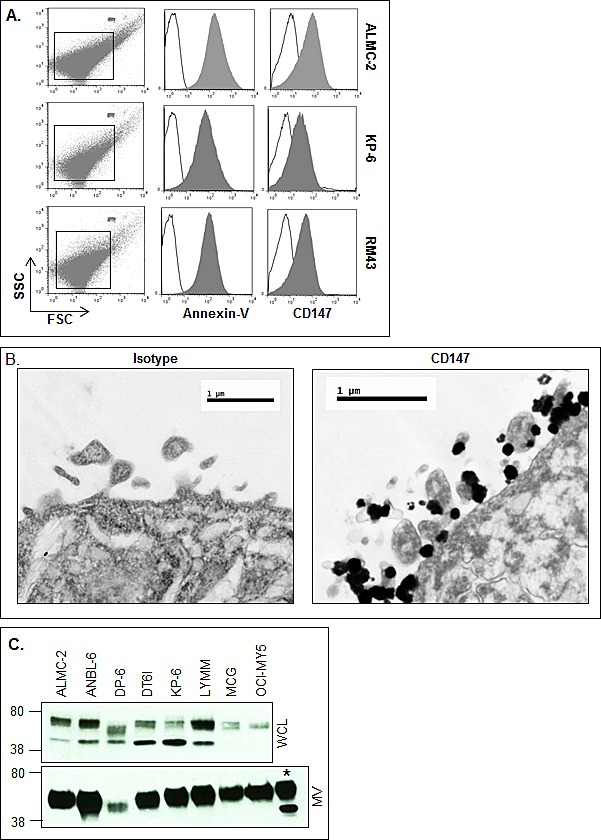
HMCLs shed MVs are enriched for CD147 A) Flow cytometric analysis of HMCL-derived MVs stained with MV marker annexin-V. CD147 staining identifies the molecule as a component within HMCL MVs (shaded histogram) as compared to the isotype control (open histogram). 1 μM beads used for size verification. B) IEM employing immune-gold particles to view CD147 expressing ALMC-2 MVs (right panel) as compared to the isotype-matched control (left panel). C) Western blotting of HMCL panel for CD147 in 5 μg MV lysates (lower panel) and whole cell lysates (WCL; upper panel). Asterisk (*) denotes ALMC-2 WCLs used as a positive control.

The images shown in Figure 2 support our conclusion that MM cells release MVs. However, MVs are also defined as being annexin-V+. Therefore, we next assessed annexin-V expression on HMCL-derived MVs by flow cytometry, and given our prior work on CD147 in MM [25] and reports in the literature that CD147 is present on MVs isolated from lung and ovarian cancer cell lines [23, 24], we also assessed MM-derived MV CD147 expression. As shown in Figure 3A, MVs isolated from 3 representative HMCLs were indeed all annexin-V+; the MVs were also clearly CD147 positive (Fig. 3A, right histograms). To further investigate MV CD147 expression, we used IEM. Figure 3B shows CD147 expression on released and cell surface associated MVs on the ALMC-2 cell line.

Because the CD147 molecule can be cleaved from cell surfaces [34], it was next necessary to characterize the molecular size of MV-associated CD147. To accomplish this, we used western blot analysis to probe the size of MV-associated CD147 vs. intact cell membrane CD147. We also used a CD147 antibody that recognizes the intracellular domain (C-terminus) thereby only permitting detection of transmembrane CD147. As we have previously shown [25], when whole cell lysates are probed for CD147, there are two dominant species observed corresponding to low and high glycosylated forms of CD147 (Fig. 3C, upper panel). In marked contrast, western analysis of MV CD147 expression revealed (Fig. 3C, lower panel) only the high glycosylated form of the molecule, known to be essential for CD147 bioactivity [35, 36].

### Immunophenotypic characterization of MM-derived MVs

**Figure 4:**
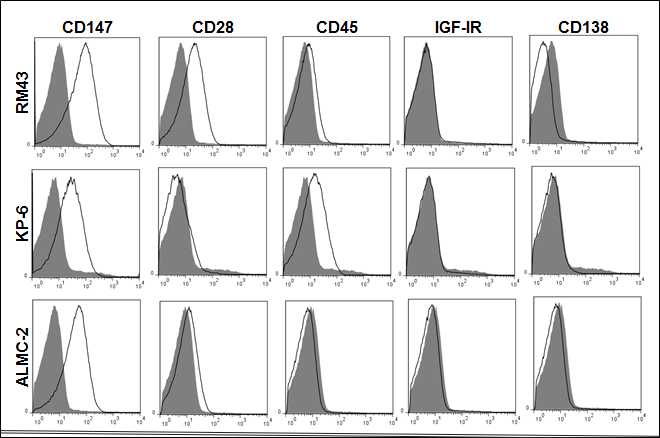
HMCL MV phenotypic analysis Annexin-V+ HMCL MVs were identified and assessed for CD28, CD147, CD138, CD45 and IGF-IR by flow cytometry (open histogram), revealing CD147 enrichment as compared to other markers in panel. Isotype (shaded histogram) used as a control.

**Figure 5:**
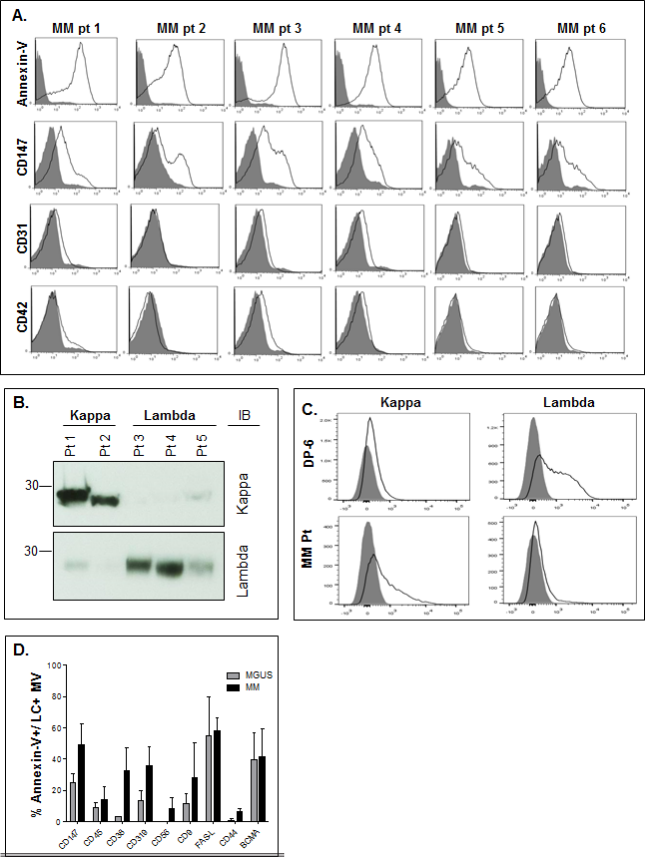
MM platelet free plasma MV characterization A) Annexin-V+ MVs harvested from MM platelet free BM plasma were assessed for CD147 levels and CD31 and CD42 (open histograms) as compared to an isotype control (shaded histogram). B) Western blotting of platelet free BM plasma MVs harvested from 2 kappa (κ) and 3 lambda (λ) MM patients identifies LCs as a component of the MVs. C) Flow cytometry of DP-6 cell line (λ–restricted) and MM pt (κ–restricted) showing positivity of the appropriate LCs in MVs (open histogram) as compared to isotype control (shaded histogram). D) Platelet free BM plasma MV content of MM patients assessed for expression of a panel of markers. Data represent the percentage of dually stained annexin-V+ and LC-restricted MVs from the patient samples which contain the indicated molecule.

We next studied if MM-derived MVs expressed additional biologically relevant receptors that are present on the surface of MM cells. Beginning with three representative HMCLs, Figure 4 again shows that MVs are enriched for CD147 expression as compared to the other molecules assayed. CD28 was present in 2 of the 3 HMCL-derived MVs as was CD45, albeit only slightly. We did not find evidence of the IGF-IR or CD138, a specific PC marker found on both normal and malignant cells, in the MVs. We next wished to characterize MVs isolated from patient plasma. In results not shown, we used Nanoparticle Tracking Analysis to determine the size and concentration of MVs found in normal and patient BM plasma. MVs harvested from normal BM and MGUS patient BM plasma contained an average of 1.1×10^9^ and 5.7×10^9^ MVs per mL, respectively. By contrast, the number of MVs isolated from MM BM plasma was approximately 4-fold higher (2.6×10^10^ MVs per mL). Although a sizeable difference was noted in the MV concentration, there was no difference in average MV size across the three patient groups. Thus, MVs from normal BM plasma were on average 101 nm, while MGUS MVs were measured at 102 nm followed by MM plasma MVs averaging 92 nm (data not shown). We next analyzed the immunophenotype of MVs from 6 representative MM patients and Figure 5A shows that they were all annexin-V+ as well as CD147+. Because platelets and endothelial cells also express CD147, it was possible that the CD147+ MVs in BM plasma derived from either or both of these cell types. However, as shown in Figure 5A, the MVs largely lacked expression of CD31 and CD42, molecules known to be present on endothelial cell and platelet-derived MVs, respectively.

We next tested the ability of tumor cell Ig light chains (LC) to specifically mark MM cell-derived MVs. Precedence for this approach was provided by findings in the literature that urinary exosomes in amyloidosis patients contain LCs restricted to the LC displayed by the malignant PCs [37]. Thus, we isolated MVs from platelet free plasma from 5 MM patients (2 patients with kappa LCs and 3 patients with lambda LCs) and performed western blot analysis. Figure 5B shows that all 5 samples had an expected 25kDa band corresponding to the LC monomer and that the MV LC signal precisely matched the patient tumor cell LC isotype. We also assessed MM patient-derived MVs by flow cytometry and Figure 5C reveals that MVs from the lambda LC-expressing DP-6 HMCL only react with anti-λ and not anti-κ antibodies whereas MVs from the representative κ-expressing MM patient sample were κ-positive and λ-negative. These results demonstrated the utility of this tool in specifically identifying patient plasma MVs that are highly likely to be released by the clonal tumor cells, thereby permitting specific phenotypic analysis of the MM-derived MVs. We next analyzed BM plasma MVs from 2 MGUS and 3 MM patients and first identified annexin-V+ MVs that also expressed the patient specific LC isotype, thus allowing us to gate on MVs exclusively derived from the PC clone. Using this approach, we were able to demonstrate that CD147 was present on average in ~50% of MM PC-derived MVs, while only 25% of MGUS LC-positive MVs contained CD147 (Figure 5D). Higher percentages of LC-restricted MVs from MM cells expressed CD38, CD319 (FAS), CD44, and CD9 as compared with MVs from MGUS patients.

### HMCL MVs induce proliferation

**Figure 6:**
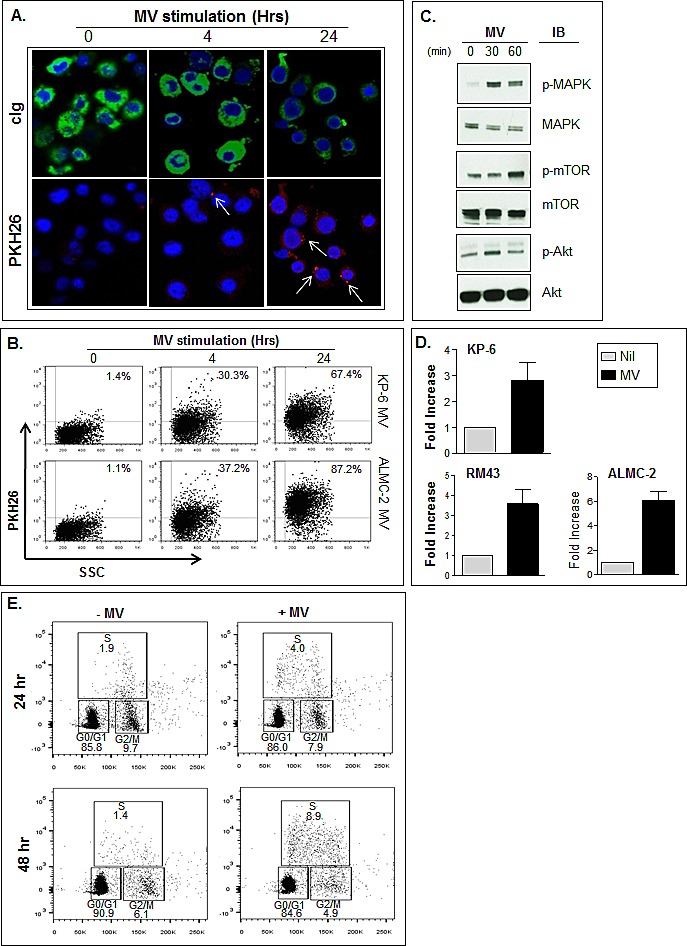
MVs promote HMCL proliferation A) Confocal microscopy revealing internalization of PKH26 stained ALMC-2 MVs (red) indicated by white arrows in RM43 cells after 4 and 24 hr (50 μg/ml). RM43 cells visualized using cytoplasmic Ig (green) and DAPI (blue) counterstain. B) Flow cytometry revealing uptake of both PKH26 stained KP-6 and ALMC-2 MVs (50 μg/ml) in RM43 cells at 4 and 24 hr time points. C) Western blot showing MV induced phosphorylation of MAPK, mTOR and Akt pathways at 30 and 60 minutes following 50 μg MV stimulation. D) HMCLs KP-6, RM43 and ALMC-2 were used to assess the activity of ALMC-2 MVs. Proliferation was assessed by ^3^H-TdR incorporation after cells were cultured for 72 hrs under unstimulated (Nil) conditions or with 50 μg/mL MVs. Results are displayed as fold increase over background. E) BrdU incorporation as shown by flow cytometry reveals HMCL LYMM transiting the cell cycle after stimulation of 50 μg/ml ALMC-2 MVs at both 24 and 48 hrs.

We next examined the biological activity of MM cell derived MVs and began using the HMCLs because of the lack of potentially confounding non-MM cells. As a first step, we determined if MVs could be internalized by the HMCLs. HMCL-derived MVs were stained with the membrane dye PKH26 prior to culturing with ALMC-2 cells for 4 and 24 hrs. Confocal microscopy (Fig. 6A) demonstrates the accumulation and uptake of MVs by cells stained for cytoplasmic immunoglobulin. Likewise, flow cytometry shows RM43 cells possess the ability to incorporate fluorescently labeled MVs derived from both KP-6 and ALMC-2 cells (Fig. 6B), with levels of uptake increasing over time. Figure 6C shows by western blot that incubation of HMCL MVs with RM43 cells at 30 minutes induced both MAPK and Akt activation while mTOR phosphorylation was observed at 60 minutes. On the basis of our findings, we next examined whether MV-induced activation of these pathways resulted in HMCL proliferation. As expected, all HMCLs tested were growth responsive to IL-6, albeit to differing degrees (data not shown). Indeed, addition of HMCL-derived MVs from common collections reproducibly increased DNA synthesis in all HMCLs. MV stimulation revealed a 2.8, 3.8, and 6-fold increase in proliferation over background of KP-6, RM43 and ALMC-2 cells respectively (Fig. 6D). Moreover, cell cycle analysis added further support that MVs enhance HMCL progression through the cell cycle (Fig. 6E).

### CD147 enriched patient plasma MVs enhance HMCL proliferation

**Figure 7:**
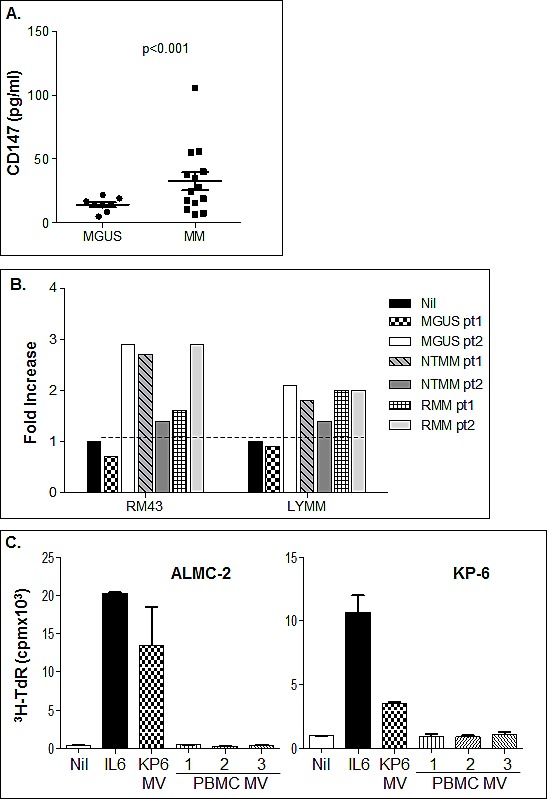
Bioactivity of MVs prepared from platelet-free bone marrow patient plasma A) BM patient platelet free plasma derived MVs from MGUS (left panel) and MM pt samples (right panel) were assessed for CD147 protein levels by ELISA. B) HMCLs RM43 and LYMM were used to assess the activity of various MV samples (50 μg/ml) from MGUS, NTMM and RMM patients. Proliferation was assessed on day 3 by ^3^H-TdR incorporation and results displayed as fold increase over background. C) HMCL MVs but not normal PBMC-derived MVs induce HMCL proliferation. ALMC-2 (left panel) and KP-6 (right panel) cells were incubated for 72 hrs in the presence of MVs (50 μg/ml) obtained from either HMCL KP-6 cells or normal donor peripheral blood mononuclear cells. IL-6 (1 ng/ml) was used as a positive control and proliferation was assessed by ^3^H-TdR incorporation.

Given that we had determined by flow cytometry that MVs obtained from MM platelet free plasma are enriched for CD147 we next sought to further confirm this finding by quantifying MV CD147 levels in a separate and distinct panel of MVs isolated from 8 MGUS and 14 MM platelet free BM plasma by ELISA. Figure 7A clearly shows that MM patients have MVs with significantly higher levels of CD147 than those obtained from MGUS samples. Although this assay does not allow us to definitively conclude that all of the detected CD147 was MM cell-derived, it does confirm our previous findings that CD147 is upregulated during disease progression and furthermore, allows us to postulate that CD147 may be a component of BM MVs in MM patients. Because of these results, we next determined whether MM patient plasma MVs also have the ability to enhance MM cell proliferation. MVs were harvested from the platelet free BM plasma from 2 MGUS, 2 never treated MM (NTMM), and 2 RMM patients. The bioactivity of these MV samples was assessed using the RM43 and LYMM HMCLs (Fig. 7B). Indeed, while one MGUS MV sample lacked activity, the second MGUS sample stimulated both HMCLs. MVs from the NTMM and RMM patients displayed variable levels of activity when assessed on the RM43 and LYMM HMCLs. Finally, to address the possibility that non-MM cell-derived MVs were stimulatory in this assay, we isolated MVs from normal peripheral blood mononuclear cells (PBMC) and compared their activity with HMCL-derived MVs. Of note, PBMCs produced on average one-tenth of the amount of MVs released by MM cells. Figure 7C clearly shows the lack of activity of PBMC MVs. Overall, these data suggest that tumor-derived MVs may be a novel mechanism by which various members of the MM clone are stimulated to proliferate *in vivo*.

### MVs promote proliferation of HMCLs through a CD147-mediated mechanism

**Figure 8:**
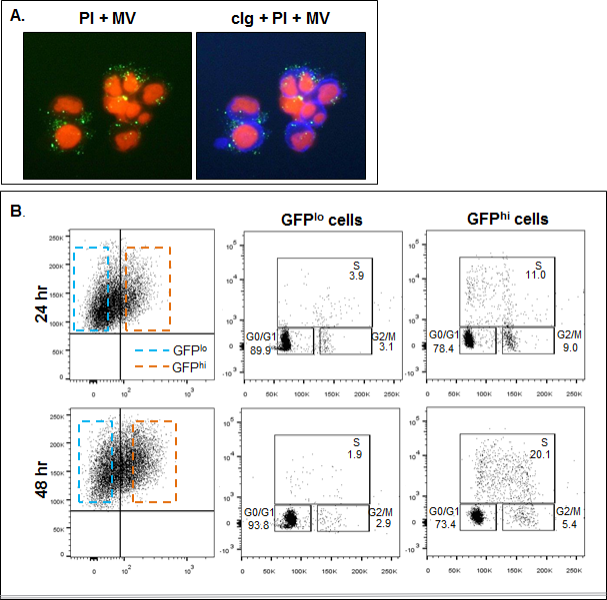
HMCL uptake of CD147 enriched MVs A) Incubation of ALMC-2 cells (blue, cIg stain; right panel) at a concentration of 2×10^6^/ml in IMDM+ 0.5% BSA with 50 μg/ml ALMC-2 derived CD147^GFP^ MVs (green) for 24 hrs revealed CD147 cellular uptake as assessed by immunofluorescence. PI was used as a counterstain. B) ALMC-2 cells were cultured at a concentration of 2×10^6^/ml in IMDM+ 0.5% BSA +/- 50 μg/ml ALMC-2 derived CD147^GFP^ MVs for 24 and 48 hrs. After incubating for 4 hrs at 37°C in the presence of BrdU, cell cycle analysis was performed. Cells incorporating higher levels of CD147 (GFP^hi^; orange dashed box) were shown to be enriched in both S and G2/M phases at both time points (right panels) as compared to cells which incorporated less CD147 (GFP^lo^; blue dashed box).

The last series of experiments were performed to determine if there is an association between MV-induced proliferation and CD147. We first transfected the ALMC-2, RM43, and KP-6 HMCLs with a CD147^GFP^ construct and MVs were collected from stably transfected cells. Figure 8A demonstrates by immunofluorescence that culturing MVs containing CD147^GFP^ with ALMC-2 cells for 48 hours resulted in cellular uptake of CD147^GFP^ MVs. Because MVs drive HMCLs through the cell cycle (Fig. 6E), to assess whether CD147 specifically contributed to this result, we incubated ALMC-2 cells with CD147^GFP^-containing MVs for 24 and 48 hrs. FACS analysis revealed CD147 incorporation by GFP fluorescence. GFP^hi^ and GFP^lo^ populations were gated and subjected to cell cycle analysis. Figure 8B shows there were significantly more GFP^hi^ cells in S phase at both time points than GFP^lo^ cells.

**Figure 9:**
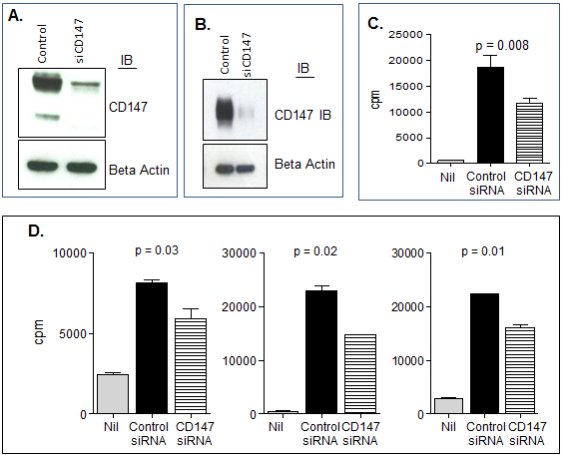
CD147 plays a role in MV bioactivity A) Western blot verifying CD147 knockdown in ALMC-2 whole cell lysates. Beta actin used as a loading control. B) Western blot confirming downregulation of CD147 in ALMC-2 derived MVs. 72 hrs after transfection of control siRNA or CD147 specific siRNA, MVs were collected. C) Downregulation of CD147 in HMCL-derived MVs as compared to mock transfected MVs decreased the proliferative response observed in ALMC-2 cells measured after 3 days of culture in IMDM+ 0.5 BSA +/− 50 μg/mL MVs. D) MVs were obtained from the KP-6 and ALMC-2 HMCLs 48-72 hours after transfection of either control or CD147 specific siRNA. Untreated KP-6 and ALMC-2 cells were then cultured in the presence of 10 μg of control or CD147 siRNA MVs. Proliferation was assessed by [^3^H]thymidine incorporation after 72 hours. Proliferation of KP-6 cells with KP-6 MVs (left panel); ALMC-2 wth ALMC-2 MVs (middle panel), and KP-6 cells with ALMC-2 MVs (right panel).

**Figure 10:**
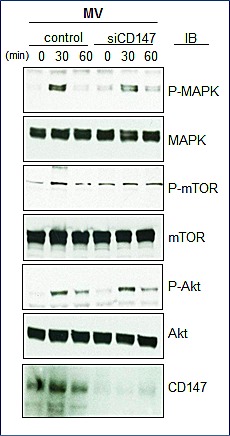
Downregulation of MV CD147 effect on signaling pathways Western blot assessing the ability of MVs isolated from cells transfected with control siRNA or CD147 siRNA to activate MAPK, mTOR and Akt pathways in ALMC-2 cells. Cells were stimulated for 30 min with 50 μg of MVs.

To further complement these studies and to strengthen our conclusion of the relationship of CD147-associated MVs and MM proliferation, we assessed the activity of MVs obtained from the HMCL KP-6 that had undergone siRNA-mediated downregulation of CD147. Figure 9A demonstrates by western blot that CD147 protein levels were successfully decreased in cell lysates as well as in MVs isolated from CD147 silenced KP-6 cells (Figure 9B). Multiple HMCLs were then cultured with MVs obtained from either control siRNA transfected cells or CD147 siRNA transfected cells and proliferation was assessed. Consistent with previous proliferation results, MVs from control siRNA transfected cells were able to enhance HMCL DNA synthesis, however, MVs collected from CD147 downregulated cells were significantly less effective (Figure 9C). Similar results were observed when MVs obtained from CD147 siRNA treated KP-6 and ALMC-2 cells were incubated with either the HMCL from which the MVs were isolated or mixed in a heterologous manner (Figure 9D). When incubating HMCLs with MVs harvested from CD147 siRNA transfected cells, western blotting revealed a decrease in MAPK and mTOR phosphorylation at 30 minutes as compared to those harvested from control siRNA transfected cells (Figure 10). AKT phosphorylation appeared unchanged. These results add support to the conclusion that the stimulatory activity of HMCL MVs can be attributed at least in part to CD147.

## DISCUSSION

Previous work in the MM field has demonstrated that the interaction of malignant PCs with BM stromal cells is essential for survival of the abnormal PC clone [5, 6]. Specifically, MM genetic alterations have been shown to modify the biology of BM stromal cells (i.e., altered adhesion molecule expression and cytokine production), which effectively conditions the microenvironment to provide optimal support of malignant PC survival and proliferation. Thus, MM is an ideal model to study the effects of the tumor microenvironment on disease progression. Notably, there is currently a growing body of literature demonstrating that important aspects of tumor microenvironment interactions stem from the dysregulated release of bioactive MVs by tumor cells. The interaction of MVs and the transfer of their bioactive content to cells in the tumor microenvironment have been shown to have profound consequences on tumor growth and metastasis. Despite the fact that numerous publications in recent years have demonstrated MV shedding from tumor cells, this area of investigation is still considered relatively novel, especially in the field of MM [11]. In the current study, we sought to determine whether primary MM patient samples and HMCLs shed bioactive MVs. Indeed, all MM patient samples and HMCLs tested were found to shed MVs. Given that shedding of MVs generally increases following activation of pathways involved in cell stimulation, stress, or oncogenesis [38], it could be speculated that the level of MV release may increase across the disease spectrum from MGUS to SMM to MM. Further studies examining whether the level of MV shedding correlates with the disease status and aggressiveness are currently underway.

In addition to our demonstration that MM cells shed MVs, we also provide provocative evidence that MM-derived MVs are internalized by MM cells and that they have a feedback effect on the MM tumor cells themselves as revealed by their ability to stimulate signaling pathways associated with cell growth and to enhance MM cell proliferation. Our observations are consistent with prior publications showing that MVs have the ability to stimulate tumor cell growth through the transfer of bioactive molecules [39-41]. In this regard, all HMCL-derived MVs were found to induce proliferation in all HMCLs tested. Both a homotypic and heterotypic approach was utilized to ensure that the proliferative potential was not limited to one cell line or from a particular harvest of MVs. While MVs obtained from platelet free BM plasma from MGUS, NTMM and RMM patients varied in their ability to induce HMCL proliferation, strong conclusions regarding these findings were not able to be drawn due to the small number of patient samples tested. However, one explanation for the variable responsiveness observed in the MGUS samples is that the MGUS platelet free BM plasma derived MVs that did not elicit a response were from a patient with stable disease status whereas the MVs from the MGUS sample that induced a proliferation response is from a patient who is beginning to progress to either SMM or MM. Serial samples are necessary to formally test this speculation and beyond the scope of the current work.

Immunoglobulin rearrangement results in PCs becoming either kappa or lambda LC restricted and malignant PCs generally produce an overabundance of free LC. Notably, we determined that MM-derived MVs contain the LC expressed by the malignant PC clone. Detection of MV-associated LC by flow cytometry suggests that the LC is potentially present on the MV surface. Although this observation seems counterintuitive given the secreted nature of free LCs, there is precedence in the literature for free LC assemblies to form on the surface of MM cells [42]. Regarding MM, the presence of LCs in MM-derived MVs is a novel finding. Additionally, this observation establishes a potentially innovative tool for identifying and monitoring malignant PC-derived MVs in patient plasma over the disease spectrum from MGUS to SMM to MM.

In prior studies [25], we demonstrated that MM cells overexpress CD147 and is involved in MM cell proliferation. In this study we extend our prior work by showing that this molecule is present in MVs released by MM cells, and that it plays a role in MV-mediated stimulation of MM cell proliferation. Thus, MVs from all MM patient samples and HMCLs were found to be enriched for CD147. Although CD147 is known to be heterogeneous in terms of its N-glycosylation status, MM-derived MVs only contained the more highly glycosylated version of CD147. This form of CD147 is the more bioactive molecule with a greater capacity to induce various biological responses such as induction of MMP production [28, 35, 43]. Using HMCLs transfected with a CD147-GFP construct, we generated HMCL MVs which contained fluorescently labeled CD147. We first showed that CD147^GFP^ MVs associated with the malignant PCs. This result was consistent with what we observed using dye-labeled MVs; however, use of the CD147^GFP^ MVs is more rigorous in testing association and internalization due to the known pitfalls using dye-labeled MVs [41]. Because of CD147 homophilic interactions [28, 44], the observed association between CD147^GFP^ MVs and the ALMC-2 cells suggested that CD147 may serve as one possible route of intercellular communication. Indeed, MM cells that internalized the greatest amount of CD147^GFP^ MVs were greatly stimulated to enter the S phase of the cell cycle, whereas MM cells that only displayed low uptake of CD147^GFP^ MVs were largely restricted within G0/G1. Moreover, when we downregulated CD147 in the HMCLs, not only was surface CD147 diminished, but CD147 contained within MVs was also greatly reduced. This reduction of CD147 within the vesicles adversely affected their ability to stimulate proliferation of the HMCLs. Taken together these results support a critical role for CD147-enriched MVs in MM cell proliferation.

Our observations that MM-derived MVs have apparent auto-stimulatory activity on MM cells are very intriguing. Although MM is a clonal PC disorder, there is well-known heterogeneity within the clonal PCs. Therefore, we speculate that MM-derived MVs may provide a means by which more proliferative members within the clone can augment the proliferation and/or survival of less proliferative tumor cells. In support of this concept, in unpublished studies (Arendt and Jelinek), we have shown that single cell cloning of MM cells gives rise to populations of cells with differing growth rates and cytokine responsiveness. Finally, it is important to acknowledge that MM-derived MVs are highly likely to have biological effects on other cell types in the microenvironment. It is also probable that the CD147 molecule in MVs contributes to this activity as well given the known ability of CD147 to enhance angiogenesis and stimulate extracellular matrix degradation [28, 29]. Indeed, studies of this nature are currently underway.

An important question that remains to be answered is whether MVs play a role in metastasis in MM patients. In patients diagnosed with the disease, it is not uncommon to observe early focal tumor involvement in MM. However, with disease progression tumor cells somehow gain the ability to thrive in distant locations either within the bone marrow or in extramedullary sites resulting in the appearance of systemic intramedullary and extramedullary lesions. It is interesting to consider the hypothesis that bone marrow-resident MM cells release MVs into the circulation thereby creating pre-metastatic niches in other anatomic locations that attract and/or nurture the expansion of metastatic PCs. Consistent with this model, circulating PCs have been described in MM patients [45] and such levels correlate with both SMM and MM disease outcome. This hypothesis is consistent with studies in other malignancies including lung, breast, melanoma, and colon cancer, where it has been shown that tumor-derived MVs have the ability to modify the local tumor microenvironment and contribute to metastatic spread of disease [46-49].

The findings described in this study significantly enhance our understanding of intercellular communication in MM by demonstrating that MM cells release biologically active MVs. Our findings that these MVs are enriched in CD147 expression and stimulate MM cell proliferation support the hypothesis that MM-derived MVs play a significant role in disease progression in the monoclonal gammopathies. Towards this end, we have begun to study MV levels and their correlation with MM disease progression as well as their ability to promote multiple biological activities that directly affect tumor cells and the tumor microenvironment including induction of angiogenesis and MMP production, and immunosuppression.

## MATERIAL AND METHODS

### Patient material

MGUS and MM patient BM aspirates were collected as part of the routine clinical examination. BM aspirates obtained from patients undergoing spine surgeries without coincident B lineage malignancies served as a source of normal BM PCs. The Mayo Clinic Institutional Review Board approved the protocol to obtain samples from healthy donors as well as from individuals with monoclonal gammopathies. Written informed consent to participate in this research study was provided by all subjects from whom BM aspirates were drawn in accordance with the Declaration of Helsinki. Normal donor peripheral blood (PB) served as the normal B cell source. BM aspirate mononuclear cells (MNCs) as well as PBMCs were isolated by Ficoll-Paque density gradient centrifugation. PCs were isolated from patient BM aspirates by magnetic bead separation using the human CD138 positive selection kit and a Robosep Cell Separator (StemCell Technologies, Vancouver, Canada).

### Cell lines and culture medium

The HMCLs ANBL-6 [50], ALMC-2 [51], KP-6 [52], DT6I, LYMM, MCG, DP-6 [52],and RM43 were all derived in our laboratory while OCI-MY5 was obtained from ATCC (Manassas, VA, USA). HMCLs were maintained in IMDM supplemented with 5% heat-inactivated FBS (PAA Laboratories, Etobiocoke, Ontario, Canada) and 1 ng/ml recombinant IL-6, generously provided by Novartis (Basal, Switzerland).

### MV culture/isolation

HMCLs and patient cells were washed in sterile saline, resuspended in IMDM+ exo-free FBS (SBI, Mountain View, CA, USA) at 2x10^6^ cells/ml, and cultured for the indicated times at 37°C. After incubation, the cell/media suspension was centrifuged at 1300 rpm for 10 minutes at 4°C to remove cells. The supernatant was then centrifuged at 2500xg for 10 minutes at 4°C, and again at 17,000xg for 45 minutes at 4°C. Collected MVs were resuspended in DPBS, centrifuged at 17,000xg 2 more times and resuspended in sterile filtered PBS before quantifying using a BCA protein assay (Pierce, Rockford, IL, USA). MM patient platelet free plasma was obtained from BM aspirates by centrifuging at 2500xg for 20 minutes at room temperature (RT) two times prior to isolating MVs.

### Immunophenotypic analysis

Cells were stained using previously described flow cytometry methods [51]. MVs were resuspended in Hanks/Hepes buffer and stained for 30 minutes at RT using various antibodies. To immunophenotype the MVs, the following antibodies were used: PE-conjugated CD147 (Abcam, Cambridge, MA, USA), PE-conjugated mouse IgG1 isotype control, VEGF R2 and IGF-IR (R&D, Minneapolis, MN, USA), FITC-conjugated kappa and lambda (BD Biosciences, San Diego, CA, USA), PE-conjugated CD138, CD45, CD38, CD28, CD44, CD9, CD56 (BD Biosciences), PE-conjugated FASL (Molecular Probes, Grand Island, NY, USA), PE-conjugated BCMA (Biolegend, San Diego, CA, USA), PE-conjugated CD319 (E-Bioscience, San Diego, CA, USA), APC- conjugated-Annexin-V (BD Biosciences), and FITC-conjugated and PE-conjugated Annexin-V (Invitrogen, Grand Island, NY, USA). After incubation, 1% paraformaldehyde was added to each sample along with the addition of 1 μM TRUCount beads (BD Biosciences, San Diego, CA, USA) (diluted in 1% PF) per manufacturer's instructions prior to analysis on BD FACSCanto flow cytometer (BD Biosciences). Sample collection was completed after acquiring 1250 TRUCount bead events. MV size was verified using a submicron bead calibration kit (Bang Laboratories, Fishers, IN, USA). Data were analyzed with FlowJo software (Tree Star, Ashland, OR, USA). MVs are defined as events ≤1 μM in diameter and positive for annexin-V.

### Nanoparticle tracking analysis

The presence, size distribution, and concentration of vesicles was assessed by nanoparticle tracking analysis using a NanoSight NS300 instrument (NanoSight Ltd., Amesbury, UK). MV samples were diluted with PBS at a range of concentrations between 4 × 10^8^ and 8 × 10^8^ particles per milliliter. Each sample was loaded into a flow-cell top-plate using a syringe pump and three videos of 30 seconds were recorded and analyzed by NanoSight software (NTA 2.3.5 B16).

### Electron microscopy (EM)

For scanning EM (SEM), samples were suspended in a fixative comprised of 4% paraformaldehyde, 1% glutaraldehyde in phosphate buffered saline, pH 7.2 (Trumps), dried through a graded ethanol series, critical-point dried, mounted and sputter-coated for 90 seconds with gold palladium. The samples were then analyzed using a Hitachi S-4700 Field Emission Scanning Electron microscope; 0-30kV (Hitachi High Technologies America, Inc., Pleasanton, CA, USA). For transmission EM (TEM), samples were fixed in Trumps fixative, scraped from ACLAR® film and placed into 2% low melting agar. Samples were stained with 1% osmium tetroxide and 2% uranyl acetate, dehydrated through an ethanol series and embedded into Embed 812 resin. Following a 24 hr polymerization at 60°C, 0.1 μM ultrathin sections were post-stained with lead citrate. Micrographs were acquired using a JEOL 1400 TEM (JEOL, Tokyo, Japan). For immunolabeling EM (IEM), samples were collected and stained with anti-CD147 antibody (Genetex, Irvine, CA, USA) for 30 min at 4°C in PBS+2% MV-depleted FCS. Cells were then washed in PBS 3X and stained with colloidal gold conjugated anti-rabbit Ig (Amersham, Piscataway, NJ, USA) for 30 min at 4°C followed by 3 washes using PBS. After the last wash, samples were silver enhanced and fixed in Trumps fixative. Samples were then processed following the TEM protocol.

### DNA synthesis assays

DNA synthesis assays measuring [^3^H]-thymidine (TdR) incorporation (Perkin Elmer, Waltham, MA) were performed as previously described [52].

### Cell cycle and DNA content analysis

HMCLs were incubated in complete IMDM media containing 10 μM bromodeoxyuridine (BrdU) overnight prior to staining the next day using a BrdU-APC flow kit (BD Biosciences) as previously described [53]. MV incorporation was analyzed and cell cycle analysis was determined on GFP^hi^ and GFP^lo^ cell populations using the FACStar (BD Biosciences). Data were analyzed using FlowJo software (Tree Star, Ashland, OR, USA).

### CD147 ELISA

CD147 was assayed by a conventional sandwich based ELISA following the manufacturer's protocol (Sinobiologicals, Bejing, China). Captured CD147 was visualized and absorbance readings were made at 450 nm, using a 96-well EPOCH microplate spectrophotometer (Biotek, Winooski, VT, USA). CD147 levels in samples were determined by interpolation from a standard curve.

### Western blot analysis

Cells lysates were subjected to 10% SDS-PAGE and CD147 probing was performed as previously described [25]. Blots were probed with one or more of the following antibodies: CD147 (GeneTex) at a 1:2500 dilution, phospho MAPK or total MAPK at a 1:1000 dilution (NEB, Ipswich, MA, USA), phospho mTOR or total mTOR at a 1:1000 dilution (Millipore), kappa or lambda (Southern Biotech, Birmingham, Alabama, USA), or β-actin (Novus, Littleton, CO, USA) at a 1:5000 dilution. HRP-conjugated secondary antibodies (GE Healthcare, Fairfield, CT, USA) were used at a 1:2000 dilution.

### Labeling of MVs and ALMC-2 with PKH fluorescence

MVs were labeled with a PKH26 red fluorescent labeling kit (Sigma-Aldrich, St. Louis, MO, USA) per manufacturer's instructions. ALMC-2 cells were stained for cytoplasmic Ig using FITC-conjugated polyclonal anti-human Ig (H+L) antibody (BD Biosciences) after fixing with 95% ethanol for 5 minutes. Slides were washed in PBS for 5 minutes prior to mounting with Vectashield containing DAPI (Vector Laboratories, Burlingame, CA, USA) and viewed by confocal microscopy (Zeiss LSM 510 confocal laser-scanning microscope, Zeiss, Thornwood, NY, USA). Images were acquired using an Olympus DP71 microscope digital camera equipped with Olympus DP manager software (Olympus Imaging America, Center Valley, PA, USA).

### CD147-GFP fusion construct

To generate the CD147-GFP fusion construct, the full-length CD147 isoform 1 (also known as Basigin-2) cDNA [54], including the 5' Kozak sequence and the ATG initiation codon but not the 3' stop codon, was amplified from cDNA synthesized from RNA isolated from the ALMC-2 cells. The forward PCR primer (5'-GGCCGCGAATTCATGGCGGCTGCGCTGTTC-3') was designed to include an *XhoI* site while the reverse primer (5'-GTCCGCCAGAGGAACTCTT CCCGGGATCCATCA-3') was engineered to include a *BamHI* site to facilitate in-frame cloning into the pEGFP-N1 vector (Clontech, Mountain View, CA, USA). The construct was validated by Sanger sequencing. To establish stable cell lines that permanently express the CD147-GFP fusion protein, the construct was linearized using the *ApaLI* restriction enzyme and then transfected into ALMC-2, RM43, and KP-6 cells by electroporation. Five days after transfection, GFP-expressing cells were sorted on an ARIA II FACS sorter (BD Biosciences). The expression of the fusion protein was validated by FACS analysis and Western blotting (data not shown).

### siRNA transfection parameters

CD147/BSG siGenome siRNA duplexes were purchased from Dharmacon Research, Inc. (Lafayette, CO, USA). Transfection of the siRNA duplexes was achieved via electroporation as previously described [25, 55]. Following the transfection period, MVs were harvested from cells as described above.

### Statistical analysis

Statistical analysis was performed using a Wilcoxon rank test. Values of *p* less than 0.05 were considered significant.
